# Fundamentals and Applications of Fluid Mechanics and Acoustics in Biomedical Engineering

**DOI:** 10.3390/bioengineering11111125

**Published:** 2024-11-08

**Authors:** Iris Little, Ephraim Gutmark

**Affiliations:** 1 Cincinnati Children’s Hospital Medical Center, Cincinnati, OH 45229, USA; iris.little@cchmc.org; 2 Department of Aerospace Engineering and Engineering Mechanics, University of Cincinnati, Cincinnati, OH 45221, USA

The field of biomedical engineering has experienced important recent advances in experimental, computational, and analytical research in fluid mechanics and acoustics. New imaging modalities, advanced instrumentation, and efficient computational methodologies have enabled these advances. Computational tools are leveraging advances in modern engineering techniques and mathematical tools. The findings contribute to a better understanding of physiological processes in the circulatory and respiratory systems, as well as phonation, and have already led to new therapies that aim to promote better human health. These transformative achievements include the early diagnosis of diseases, monitoring of their progression, individualized interventions, surgical planning, effective drug delivery, and new medical devices.

Synergy between computational tools and experimental diagnostics is important for the development of both experimental and computational approaches. Accurate experimental data are crucial for validation of Computational Fluid Dynamics (CFD) and for tuning each step of the CFD process to obtain reliable results. CFD can help in designing the experimental setup and provide a deeper understanding of experimental results by adding information that cannot be measured.

This Special Issue focuses on original research papers and on comprehensive reviews on the “Fundamentals and Novel Applications of Fluid Mechanics and Acoustics in Biomedical Engineering”. It highlights recent developments in these areas and identifies directions and topics that require further scientific research.

Manuscripts for this Special Issue were solicited from a large group of the top researchers in this field. Submissions to this thematic issue were subjected to a rigorous peer-review process following the standards and policies of the *Bioengineering* journal. Each paper was reviewed by several internationally recognized experts in the field. Thirteen papers were eventually accepted following revisions, resulting in the high standards of this issue.

The 13 papers included in this issue are grouped into two categories. The first group includes seven contributions covering upper airway flow, phonation, sleep apnea, and nasal flow; the remainder are concerned with cardiovascular flow and blood rheology.

**Airway aerodynamics and acoustics: OSA, phonation, and rhinology.**

Seven papers in this issue deal with the impact of flow and acoustics of laryngeal dynamics and phonation on Obstructive Sleep Apnea (OSA), and on rhinology.

Kraxberger et al. [1] presented an experimental and computational study of the impact of supraglottal vocal tract length on the vibrations of the silicone vocal folds model. They showed that when the vocal tract length is increased, it results in lower acoustic eigenfrequencies. When these frequencies are near the mechanical eigenmodes of the folds, the vibration frequency of the folds will align with the acoustic frequency. Conversely, when the vocal tract length is decreased, the fold vibration frequency is uncoupled with the acoustic modes. This indicates that for certain conditions, the length of the folds can influence the fold vibration frequency and therefore impact phonation.

Further support for the Kraxberger et al. findings was provided by Näger et al. [2], who performed flow field measurements using Particle Imaging Velocimetry (PIV) in the same experimental model. The measured velocity fields were used to calculate the acoustic source terms. They observed a strong interaction between the fundamental acoustic resonance frequency and the vocal fold vibration frequency when the acoustic resonance frequency was not too high. At low acoustic frequencies, the vocal fold vibrations matched the fundamental acoustic modes. The supraglottal aerodynamic pulsating frequency matched that of the folds’ vibrations. When such coupling occurs, the vocal efficiency, signal-to-noise ratio, harmonics-to-noise ratio, and cepstral peak prominence increase. This led to the conclusion that under such conditions it is possible to phonate longer and with higher quality. The range of the vocal lengths tested in this study was limited to frequencies associated mostly with children and female singers.

While the synthetic vocal folds tested by Kraxberger et al. [1] and Näger et al. [2] were cast from a single layer of silicone, Tur et al. [3] aimed to more closely represent the function of physiological larynges by adding artificial ligament fibers to a multi-layer silicone larynx model. The impact of elongation, abduction, and adduction on the laryngeal dynamics was assessed and compared to results of ex vivo and in vivo tests. The models replicated the vibration fundamental frequency, subglottal pressure, symmetry, and glottal gap. They also demonstrated the impact of various ligament parameters. This demonstrated the ability to replicate the phonation behavior of professional female singers.

A different aspect of the aeroelastic influence of the glottal flow on the folds’ dynamics is elucidated by Jiang et al. [4] They used flow-structure-interaction (FSI) simulations to investigate the impact of the laryngeal flow separation vortices (FSVs) on the folds’ vibrations. Their simulations showed that during the closing phase of the cycle, the larynx acquires a divergent shape that causes the flow to separate from the folds, generating recirculating flow between the jet and the larynx walls (called FSVs). These vortices produce negative pressure of nearly 30% of the subglottal pressure. This increases the aeroelastic energy transfer from the airflow to the vocal folds by 32%. They quantified the contribution of the FSV by comparing the impact of their presence to the case when they are artificially removed from the flow field. They showed that the vibration amplitude and flow rate were increased by 20% and the closing speed, skewness quotient, and Maximum Flow Declination Rate (MFDR) by up to 40% when FSVs were present. Their results demonstrate the importance of FSVs on vocal fold dynamics. The authors emphasize that since their model is two-dimensional, the impact of FSVs on a realistic three-dimensional model will be lower.

An application of FSI simulations for studying Obstructive Sleep Apnea (OSA) and snoring is described by Li et al. [5] They studied the dynamic response of the uvulopalatal flexible structures to respiratory flow using a simplified 3D model of the soft palate. The study included three levels of flow rate and changes in the ratio between the oral and nasal flow rates. High flow rates resulted in vortex shedding behind the soft palate that led to high-amplitude tip displacement vibrations, especially when the oral and nasal flow rates were equal or when only nasal flow was present. The large deformation was attributed to pressure differences between the oral and nasal sides of the palate. Low and medium flow rates had small wake fluctuations and low tip displacement.

In a related study, Palomares et al. [6] investigated the impact of snoring associated vibro-acoustic loading from vibrating soft tissue, catecholamine exposure, and hypoxia associated with OSA on platelet activation. They hypothesized that these factors could result in an increased risk of thrombotic stroke associated with OSA and snoring. They exposed platelets to increased sound intensity and duration and showed that a low frequency of 200 Hz had higher impact on platelet activation than higher frequency of 900 Hz. They also showed increased platelet activation by epinephrine (increased catecholamines) and hypoxia. Aspirin, which inhibits platelet activation, had no added effect on these observations.

OSA, and treatment of OSA, such as continuous positive airway pressure (CPAP), are affected by the nasal flow and nasal resistance. A comprehensive summary of the status of the computational versus experimental approaches to upper airway flow investigation is provided in Johnsen’s [7] review of nasal airflow. The paper describes the field of computational rhinology, reviews the published literature on in vitro and in silico nasal air flow, and presents results on Large Eddy Simulations (LES) computational rhinometry research. The paper also analyzes the significant disagreement between computations and in vivo rhinomanometry (RMM) data. Three possible CFD modeling deficiencies are rejected, namely, the wrong choice of the turbulent model, poor special or temporal resolution, and ignoring transient effects. Other potential reasons that could include airway tissue compliance or nasal hair effects should be considered.

**Cardiovascular flows and blood rheology.**

The other six papers of this issue discuss the impact of the aortic valve morphology on its function, the rheological properties of blood, and the possible effect of hemodynamic forces on cardiovascular disease.

An FSI computational study by Sundström and Tretter [8] investigated the impact of bicuspid aortic valve (BAV) commissural angle on valve function and proximal aortic hemodynamics. Their simulations showed that with an asymmetric commissural angle of 120°, the aortic opening area is reduced, and the ejected flow is swirling and recirculating, resulting in high wall shear stresses on the proximal ascending aorta. In a more symmetric commissural angle of 180°, these patterns are less pronounced. The asymmetry may thus lead to increased aortic dilatation and valvular deterioration, highlighting the clinical importance of considering the commissural angle.

The clinical importance of FSI assessment of aortic flow in patients with bicuspid aortic valve (BAV) led Sundström and Laudato [9] to test a Machine-Learning (ML) algorithm that will accelerate the process of segmenting a patient-specific four-dimensional phase-contrast magnetic resonance imaging (4D-PCMRI) of the thoracic aorta. They used the imaging of six subjects, three with non-stenotic tricuspid aortic valves (TAV) and three with non-stenotic functionally bicuspid aortic valves (BAV). Using TotalSegmentator-based segmentation they compared the flow field features of the two groups. They showed strong swirling motion in the proximal ascending aorta of the TAV cases compared to BAV, resulting in higher tangential shear stresses.

Sundström et al. [10] described the use of blood speckle imaging (BSI) based on echocardiographic data to compare pre- and post-operative flow patterns following subaortic membrane resection and aortic valve repair. The data indicated that the flow had less regurgitation following surgery, resulting in changes in the wall shear stresses. While the time-averaged value of wall shear stress (TAWSS) remained unchanged, the oscillatory shear index (OSI) was reduced. This indicated lower risk for aortic wall and leaflet damage.

The role of shear stresses on arterial diseases, such as atherosclerosis, was also investigated in the carotid artery by Wild et al. [11]. They developed carotid artery bifurcation models with “healthy” and “predisposed” geometries and used CFD to compare between the resulting two flow fields. They showed that in the “healthy” geometry, a hairpin vortical structure develops in the internal carotid artery (ICA) sinus and persists during a significant part of the cardiac cycle. This structure appears earlier in the cycle in the “predisposed” geometry and persists for a much shorter duration, followed by less organized structures. This change in flow behavior results in lower wall shear stresses (WSS) and a weaker favorable streamwise pressure gradient in the “predisposed” geometry, making it more prone to atherosclerotic plaque formation.

Computations of hemodynamics in the large blood vessels, such as the aorta, assume that the blood behaves as a Newtonian fluid. Others use different rheological models to represent non-Newtonian behavior. Fuchs et al. [12] compared three non-Newtonian models: Casson, Quemada, and Walburn–Schneck to Newtonian viscosity flow in the human thoracic aorta. They compared several features of the flow: (i) magnitude of the viscosity relative to the Newtonian case; (ii) wall shear stress (WSS); (iii) WSS-related quantities, Oscillatory Shear Index (OSI), Time-Averaged WSS (TAWSS) and Relative Residence Time (RRT); (iv) size of retrograde flow, when blood flows backwards; and (v) transport of small particles in the thoracic aorta. The main differences were in instantaneous WSS at low shear rates (near walls or stagnation zones), where space-averaged WSS differed by ~10% and the temporal derivative of WSS by up to 20%. Transport of particles was also impacted.

The non-Newtonian rheological properties of blood are the result of the elastic behavior of the red blood cells (RBC). Jafarinia et al. [13] proposed that since the electrical conductivity of blood is related to hemodynamics, it can become a safe, low-cost diagnostic method. They developed two anisotropic electrical conductivity models to describe two three-dimensional flows: a straight, rigid pipe and an idealized aorta geometry. In the rigid pipe, the two models matched experimental results. In the simplified Aorta model, the two models gave different results. Due to the lack of experimental data, it is not possible for now to ascertain the accuracy of the two models.

This Special Issue highlights the growing interest in flow and acoustic phenomena related to human biology and health and will encourage further scientific contributions and discussions on the fundamental physics and applications of this new and exciting topic.
